# Experimental and modeling studies on microwave-assisted extraction of mangiferin from *Curcuma amada*

**DOI:** 10.1007/s13205-013-0125-5

**Published:** 2013-04-21

**Authors:** Jeke Kullu, Abhishek Dutta, Denis Constales, Surabhi Chaudhuri, Debjani Dutta

**Affiliations:** 1 Department of Biotechnology, National Institute of Technology, Durgapur, 713209 India; 2 Department of Chemical Engineering, Groep T-Leuven Engineering College (Associatie KU Leuven), A. Vesaliusstraat 13, 3000 Leuven, Belgium; 3 Vakgroep Wiskundige Analyse, Ghent University, Galglaan 2, Blok S22, 9000 Ghent, Belgium

**Keywords:** Microwave-assisted extraction, Mangiferin, Antioxidant activity, ANOVA, Mathematical modeling

## Abstract

Mangiferin, a bioactive compound having potent nutraceutical, strong antioxidant and pharmacological significance has been extracted using microwave-assisted extraction (MAE) technique from *Curcuma amada*, commonly known as mango ginger. The extraction solvent ethanol is eco-friendly, nontoxic and reduces the risk of environmental hazards. The influence of several independent variables such as microwave power, ethanol concentration, extraction (irradiation) time and pre-leaching time has been studied under varying conditions using one-factor-at-a-time analysis to obtain an optimal extraction ratio. The maximum mangiferin content of 1.1156 mg/g is obtained at microwave power of 550 W and extraction time of 50 s with 80 % ethanol as a solvent and pre-leaching time of 20 min. The results indicate that microwave power and ethanol concentration have the most significant effect on the yield of mangiferin content. The presence of mangiferin in final *Curcuma amada* extract is confirmed through high-performance liquid chromatography and the functional groups are identified through Fourier transform infrared spectroscopy analyses using standard mangiferin. The experimental profiles are fitted into a two-parameter modified first-order kinetic model and a three-parameter modified logistic model and checked using the goodness-of-fit criterion. The *Curcuma amada* retained its antioxidant activity after MAE treatment and the antioxidant activity of mangiferin obtained after extraction using DPPH free radical scavenging assay is studied.

## Introduction

Traditional plant spices, similar to fruits and vegetables, are known to contain health-promoting components such as vitamins, minerals, antioxidants and prebiotics (Omenn et al. 1996). In particular, plant spices are used in foods because they impart desirable flavours and may fulfil more than the one function for which they are added. Extensive research is being conducted on traditional medicines, on different plant species and their therapeutic applications all over the world. *Curcuma amada*, commonly known as mango ginger, is an important member of the Zingiberaceae family. It has an Indo-Malayan origin and is distributed widely in the tropics from Asia to Africa and Australia (Sasikumar 2005). *Curcuma amada* is named mango ginger because it is morphologically similar to ginger and imparts a mango flavour and is typically used in the manufacture of pickles, culinary preparations and salads for flavour, candy and sauce (Shankaracharya 1982). *Curcuma amada* has pharmacological significance for a variety of ailments. Therapeutically, mango ginger is used to treat a range of mood and medical disorders in traditional and ayurvedic medicine. *Curcuma amada* is credited with diverse bioactive molecules demonstrating antibacterial, antifungal, anti-inflammatory, anti-hypercholesterolemic, insecticidal, aphrodisiac, antipyretic and antioxidant properties (Singh et al. 2010). Mangiferin is an important bioactive constituent of mango ginger containing xanthone-C-glycoside, which has numerous pharmacological properties and is an important phytochemical. It has antidiabetic, cardioprotective, immunomodulatory, antioxidant, antitumour, hepatoprotective and vasorelaxant properties and is useful in the treatment of biliousness, skin diseases, bronchitis, asthma and inflammation (Jatoi et al. 2007). Extraction forms the first basic step in medicinal plant research because the preparation of crude extracts from plants is the starting point for the isolation and purification of chemical constituents (Romanik et al. 2007). Keeping in mind the requirements such as shortened extraction time, reduced solvent consumption, increased pollution prevention and the special care needed for thermolabile constituents, numerous extraction techniques have been developed for the purpose of obtaining pharmacologically active compounds from various plant sources such as supercritical fluid extraction (SCFE), microwave-assisted extraction (MAE), ultrasound-assisted extraction (UAE) and heat reflux extraction (HRE). However, because of several disadvantages with the traditional extraction techniques like sonication and Soxhlet extraction, non-conventional extraction techniques like SCFE, extraction by microwave and ultrasound sources have gained importance. The use of microwaves in analytical sciences is not new; the first reported analytical use for microwave oven was in 1986 for the extraction of organic compound (Dean 2010). In recent years, MAE has attracted growing interest as it allows rapid extraction of solutes from solid matrices, with extraction efficiency comparable to that of the classical techniques (Camel 2000). Heating occurs in a targeted and selective manner in MAE with practically no heat being lost to the environment, and the mechanism can significantly reduce the extraction time (Huie 2002). This means it requires less solvent volume and is thus time conserving with improved product recovery. Further, the extraction solvent used is usually water or ethanol, which is inexpensive, nontoxic and environmentally benign (Ferguson et al. 2012). Samples pretreated with solvents with higher microwave absorbing capacity when coupled with extracting solvents like ethanol bring about heating by at least two competing mechanisms, namely direct heating from the interaction of microwaves with ethanol and heating from the diffusion of excess heat resulting from the interaction of the microwaves with the pretreated matrix (Mandal et al. 2007). In our previous study (Padmapriya et al. 2012), MAE of mangiferin from *Curcuma amada* was studied using only two independent factors, namely microwave power and extraction (irradiation) time. However, it has been observed that several other extraction variables such as solvent concentration, ethanol concentration and pre-leaching time could also be influential factors in the optimization of the extraction protocol of a bioactive compound, which may act dependently or independently (Dhobi et al. 2009). In the present study, therefore, a more rigorous approach has been applied to understand the influence of these independent factors on mangiferin extraction using mathematical modeling. The presence of mangiferin in final *Curcuma amada* extract was confirmed using high-performance liquid chromatography (HPLC) using standard mangiferin and was further subjected to Fourier transform infrared spectroscopy (FTIR) analysis for identification of the functional groups. The antioxidant activity of mangiferin obtained after extraction using DPPH free radical scavenging assay has also been studied.

## Materials and methods

### Plant material

Fresh and healthy *Curcuma amada* (mango ginger) were purchased from the local market in Durgapur, West Bengal. The rhizomes were washed, peeled and cut into fine pieces and then dried in a hot-air oven (OVFU) at 70 °C until constant weight and was well blended. Mangiferin standard was purchased from Sigma-Aldrich, USA.

### Microwave-assisted extraction (MAE)

Microwave-assisted extraction was performed using a microwave apparatus (Samsung Trio, Model CE117ADV; 230 V ~50 Hz) in a closed vessel system. 2.5 g of dried *Curcuma amada* powder was extracted with 25 ml solvent under different MAE conditions. After extraction, the vessels were allowed to cool at room temperature before opening. Microwave power (250, 350, 450, 500, 550 and 900 W), ethanol concentration (50–100 %, v/v), extraction time (1–120 s, with an interval of 5 s) and pre-leaching time (1–30 min, with an interval of 5 min) were evaluated for the extraction of mangiferin from *Curcuma amada*. The extraction of mangiferin was carried out using the method of Padmapriya et al. (2012). The final extract was evaporated and dissolved in DMSO before UV–vis spectrophotometric (Techcomp, UV 2310) analysis. For the estimation of mangiferin, the method described by Joubert et al. (2008) was used and the absorbance was measured at 410 nm.

### High-performance liquid chromatography (HPLC) analysis

The final extract of *Curcuma amada* was analysed by HPLC (Waters 600) equipped with a UV–vis detector (Waters 2489) according to the method described by Muruganandan et al. (2002). Chromatographic separation was performed on a reverse-phase column (C18, 4.6 × 250 mm, Waters) with the temperature of the column being maintained at 25 °C. The mobile phase was acetonitrile and 3 % acetic acid in the ratio 16:84 at a flow rate of 0.5 ml/min. The sample injection volume was 10 μl. The peaks were evaluated based on their absorbance at 254 nm. Retention time and concentration of the samples were compared with pure standard of mangiferin (Sigma-Aldrich, USA).

### Fourier transform infrared spectroscopy (FTIR) analysis

The mangiferin extracted after MAE at 550 W was further subjected to FTIR analysis for identification of the functional groups. Comparing the functional groups present in standard mangiferin, the damaged functional group of the extracted mangiferin can be identified. A known weight of the final sample extract was mixed with potassium bromide and loaded onto a Perkin Elmer instrument. The samples were scanned in model spectrum-100 system in range of 400–4,000 cm^−1^. The spectral data obtained were compared with a standard mangiferin chart to identify the functional groups present in the sample.

### DPPH radical scavenging activity

The DPPH assay was carried out according to the method reported by Ara and Nur (2009). DPPH solution (0.004 % w/v) was prepared in 95 % methanol. The stock solution was diluted to final concentration of 1, 5, 10, 20, 40, 60, 80 and 100 μg/ml . The freshly prepared DPPH solution was added in each of the test tubes containing the final concentrations of *Curcuma amada* methanolic extract, and after 10 min of incubation the absorbance was taken at 517 nm using a spectrophotometer. The scavenging effect (%) of DPPH free radical was measured using the following equation:

### Statistical analysis

The screening of the variables has been done using one-factor-at-a-time (OFAT) analysis, which has several advantages such as run size economy, fewer level changes and providing protection against the risk of premature termination of experiments (Qu and Wu 2005). It must be noted that although processes are commonly optimized in most industrial experiments using OFAT design approach, optimal conditions or interactions between variables cannot be predicted with this methodology (Wardhani et al. 2010). However, OFAT design allows to find out more rapidly whether a factor has any effect and is therefore a sequential learning process (Morgan and Deming 1974). The statistical software Graphpad Prism v5.0.0.2 was used for the data analysis. A two-way analysis of variance (ANOVA) was implemented to calculate the significance of the differences in the content of mangiferin. Means and coefficients of variance were computed for all qualitative analysis and treatments with homogeneous means ranked using the Newman–Keuls post hoc test. The significance of the results was established at values greater than 0.05 in all the experiments performed. The parameters of the empirical models were fitted with a nonlinear least-squares (NLLS) Marquardt–Levenberg algorithm, using the device-independent plotting program Gnuplot.

### Mathematical modeling of mangiferin extraction

Kinetics of MAE of mangiferin is performed at the experimental design points for the three independent variables namely, microwave power, ethanol concentration and pre-leaching time. In all these cases, the experimental data seem to follow a sigmoidal curve for which a two-parameter modified first-order kinetic model (Wardhani et al. 2010) and a three-parameter delayed logistic model with a final asymptote (Yukalov et al. 2009) are chosen to describe the evolution of microwave-assisted mangiferin extraction given as follows:1and2where *Y* is the mangiferin content (mg/g) at time *t*, *Y*_max_ is the maximum mangiferin content (mg/g) when time approaches infinity, *k*_m_ is the first-order mangiferin extraction constant or the specific rate of mangiferin concentration (s^−1^) and *τ* is the time delay (s); else the value at *t* = 0 would always be half the value at *t* = ∞ (and there is no sufficient reason to assume such a restriction).

A generalized expression to describe the dependency of both microwave power (*P*) and ethanol concentration (*E*) on extraction time can be written using a slightly modified delayed logistic model as follows:3where *P*_ref_ (or *E*_ref_) is the parameter related to microwave power (or ethanol concentration), respectively, and where the mangiferin yield *Y* is twice the initial value.

It is important to clarify that the extraction process is the result of an interaction between *Curcuma amada* (mangiferin) and ethanol, causing the kinetic dependence to be of the second order. During extraction, concentration of mangiferin (solute) increases and goes to saturation although it is not yet extracted. However, if excess ethanol (solvent) is added to the solution, the extraction of mangiferin occurs. This reduces the apparent kinetic dependence from a second-order rate equation to a pseudo*-*first order rate equation (see Eqs. 1, 2).

## Results and discussions

**Fig. 1:**
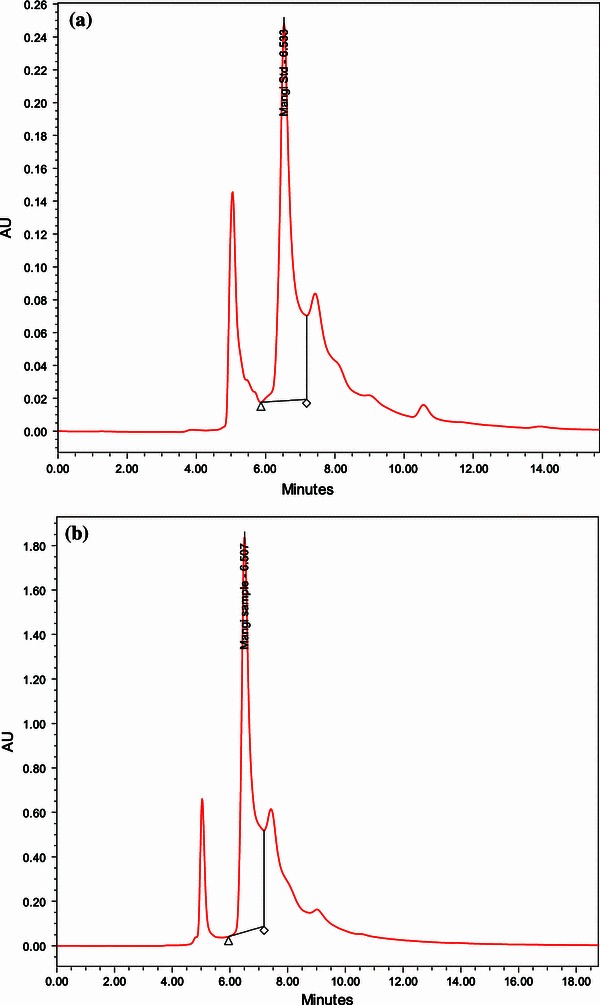
**a** HPLC chromatogram of mangiferin standard. **b** HPLC chromatogram of mangiferin extracted from *Curcuma amada* by microwave-assisted extraction (MAE)

Figure 1a, b shows the chromatographic profile of the mangiferin from *Curcuma amada* after MAE and standard mangiferin, respectively. The retention time of 6.51 min obtained from the extract agreed well with the standard verifying the presence of mangiferin in *Curcuma amada* extract.

### Effect of microwave power

**Fig. 2:**
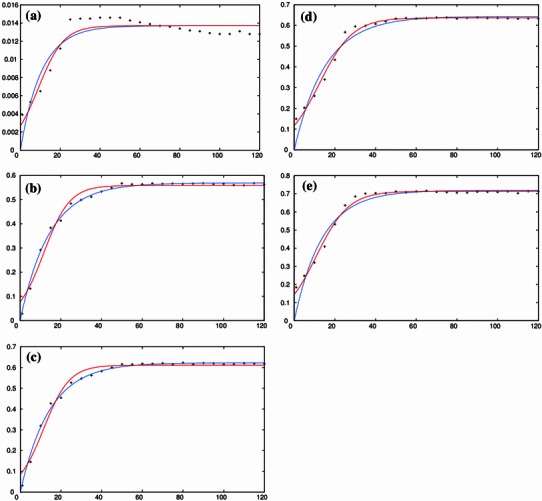
Temporal evolution of the effect of microwave power (as an independent variable) on the yield of mangiferin content extracted from *Curcuma amada* at various experimental design points (*x* axis: time in s; *y* axis: yield in mg/g). The experimental data (*symbols*) fitted to the 2-parameter model (Eq. 1, *blue*) and 3-parameter logistic model (Eq. 2, *red*) are shown in Fig. 2a–e for 250, 350, 450, 500 and 550 W, respectively

Figure 2a–e shows the effect of microwave power on mangiferin content with extraction time; the symbols represent the experimental data while the continuous curves represent the model fit. It can be clearly seen in these figures that there is a steady increase in mangiferin content up to 50 s at the power range from 250 to 550 W after which it reaches a threshold value. However, at 900 W there is a significant decrease in the mangiferin content and the yield decreases drastically, as was observed by the response at 900 W (not shown). This is in accordance with the observations of our previous study (Padmapriya et al. 2012) where a similar response was obtained for 600 W. The mangiferin content of *Curcuma amada* in the control sample, i.e. before MAE, was 0.0046 mg/g. The mangiferin content at 250 and 550 W after 50 s of microwave extraction is found to be 0.0146 and 0.7161 mg/g, respectively. This manifold increase in mangiferin content is maximum (more than 150 times higher than the control sample) at 550 W after 50 s of extraction compared with 250 W (around three times higher than the control sample) for the same extraction time. This accelerated extraction of mangiferin by increasing microwave power can be correlated to the direct effects of microwave energy on molecules by ionic conduction and dipole rotation which result in power dissipated in volumetric basis inside the solvent and plant material which generate molecular movement and heating. Microwave irradiation energy disrupts the bonds because of microwave-induced dipole rotation of molecules and migration of dissolved ions. Microwave irradiation energy can enhance the penetration of solvent into the matrix and deliver efficiently to materials through molecular interaction with the electromagnetic field and thus offer a rapid transfer of energy to the solvent and matrix, allowing the dissolution of components to be extracted. The steep decrease in mangiferin content at 900 W is due to the rapid degradation of mangiferin at higher microwave power range. As the experiments are conducted in dry matter, as is usually the case (Mandal et al. 2007), chances of degradation due to drying or evaporation at a higher microwave power intensity are ruled out. Similar results of decrease in extraction yield of astragalosides from *Radix astragali* at high power due to disorderly molecular interactions have been reported in the optimization study of MAE of four main astragalosides in *Radix astragali* (Yan et al. 2010).

Results of a two-way ANOVA with extraction time and microwave power as independent variables are given in Table 1. The mangiferin content in *Curcumaamada* is significantly dependent on microwave power and extraction time as well as their interaction. Newman–Keuls test suggest that the mangiferin content is significant at 550 W, validating our experimental results of extracting the highest mangiferin content at 550 W from *Curcuma amada*. Student’s independent *t* test further confirms that both microwave power and extraction time have a significant effect on the mangiferin content.

**Table 1:** Test of between-subjects effects: a two-way analysis of variance with microwave power and extraction time as independent factors

Source		Type III sum of squares	*df*	Mean square	*F*	Sig.	Noncent. parameter	Observed power^a^
Microwave power	Hypothesis error	10.067	5	2.013	316.937	0.0	1,584.685	1.0
		0.762	120	6.353E–03^b^				
Extraction time	Hypothesis error	1.464	24	6.101E−02	9.604	0.0	230.504	1.0
		0.762	120	6.353E–03^b^				
Microwave power × extraction time	Hypothesis error	20.906	1	20.906	342.642	0.0	342.642	1.0
		1.464	24	6.101E–02^c^				

Dependent variable: concentration of extracted mangiferin

^a^Computed using alpha = 0.05

^b^MS (Error)

^c^MS (TMIN)

### Effect of extraction time

**Fig. 3:**
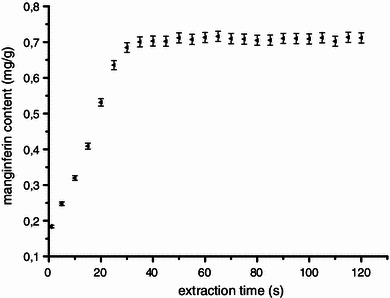
Influence of extraction time on the yield of mangiferin content. Extraction condition: pre-leaching time—1 min, microwave power—550 W and ethanol concentration—100 %. The results are expressed as means of yield ± SD

As seen in Fig. 3, mangiferin content increases significantly with the increase in extraction time from 1 to 50 s before reaching a steady state. The mangiferin content of *Curcuma amada* kept in a pre-leaching time of 1 min and extracted at 550 W for 50 s is found to be maximum around 0.7121 mg/g. Beyond 50 s of extraction time, no significant increase in mangiferin content is observed. Similar observations are also reported for MAE of artemisinin in from *Artemisia annua* (Pan et al. 2007) and tanshinones from *Salvia Miltiorrhiza* Bunge (Hao et al. 2002).

### Effect of solvent concentration

**Fig. 4:**
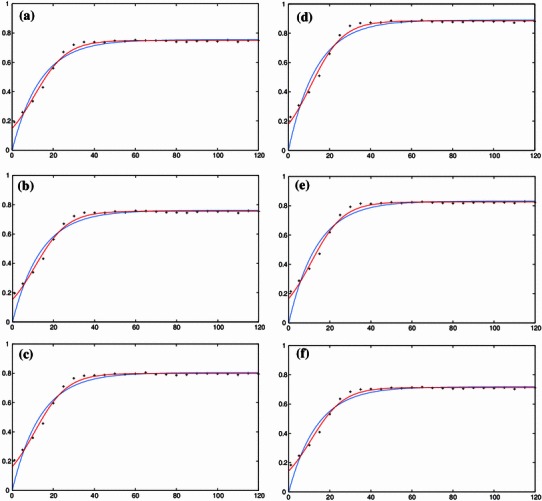
Temporal evolution of the effect of ethanol concentration (as an independent variable) on the yield of mangiferin content extracted from *Curcuma amada* at various experimental design points (*x* axis: time in s; *y* axis: yield in mg/g). The experimental data (*symbols*) fitted to the 2-parameter model (Eq. 1, *blue*) and 3-parameter logistic model (Eq. 2, *red*) are shown in Fig. 4a–f for 50, 60, 70, 80, 90 and 100 %, respectively

Preliminary screening experiments (not reported in this study) with different organic extraction solvents such as acetone, acetonitrile, methanol and ethanol have been carried out and it was observed that ethanol yielded significant mangiferin content. Ethanol undergoes less microwave absorption than water due to its lower dielectric loss value but the overall heating efficiency for the solvent will remain higher than that of water due to increased value of the dissipation factor. Extraction with aqueous ethanol has been reported in earlier studies since it has less restrictions in food applications (Wardhani et al. 2010; Wang et al. 2010; Hemwimon et al. 2007). MAE of 2.5 g of dry *Curcuma amada* powder is carried out at microwave power of 550 W, pre-leaching time of 1 min and irradiation time of 1–120 s with aqueous ethanol as solvent. The effect of aqueous ethanol concentration on mangiferin content can be seen in Fig. 4a–f. The mangiferin content increases significantly with increase in ethanol concentration up to 80 % ethanol concentration; beyond 80 % ethanol concentration there is a decrease in mangiferin content. Dhobi et al. (2009) found similar results in their work related to optimization of MAE of bioactive flavonolignan-silibinin. A maximum mangiferin content of 0.8864 mg/g is obtained in 80 % ethanol concentration at extraction time of 50 s. One possible reason for the increased efficiency with 80 % ethanol might be due to the increase in swelling of plant material by presence of some amount of water, which increased the contact surface area between the plant matrix and the solvent. Presence of some amount of water can also increase the mass transfer process by increasing the relative polarity of the solvent, thus improving its solubilizing capacity. Similar results were reported by Li et al. (2004) during microwave-assisted solvent extraction and HPLC determination of effective constituents in *Eucommia ulmoides* Oliv.

Statistical results indicate that the mangiferin is positively correlated but insignificant with the ethanol concentration but significant with extraction time. The results of ANOVA are given in Table 2. Both the ethanol concentration and extraction time along with their interaction are significant with respect to the mangiferin content. Newman–Keuls test show that the mangiferin content is significant at higher ethanol concentration of 70, 80, 90 and 100 % with ethanol concentration of 80 % yielding the highest mangiferin content.

**Table 2:** Test of between-subjects effects: a two-way analysis of variance with ethanol concentration and extraction time as independent factors

Source		Type III sum of squares	*df*	Mean square	*F*	Sig.	Noncent. parameter	Observed power^a^
Ethanol conc.	Hypothesis error	0.373	5	7.455E−02	392.693	0.0	1,963.466	1.0
		2.278E−02	120	1.898E–04^b^				
Extraction time	Hypothesis error	4.499	24	0.187	987.452	0.0	23,698.838	1.0
		2.278E−02	120	1.898E–04^b^				
Ethanol conc. × extraction time	Hypothesis error	72.827	1	72.827	388.509	0.0	388.509	1.0
		4.499	24	0.187^c^				

Dependent variable: concentration of extracted mangiferin

^a^Computed using alpha = 0.05

^b^MS (Error)

^c^MS (TMIN)

### Effect of pre-leaching time

**Fig. 5:**
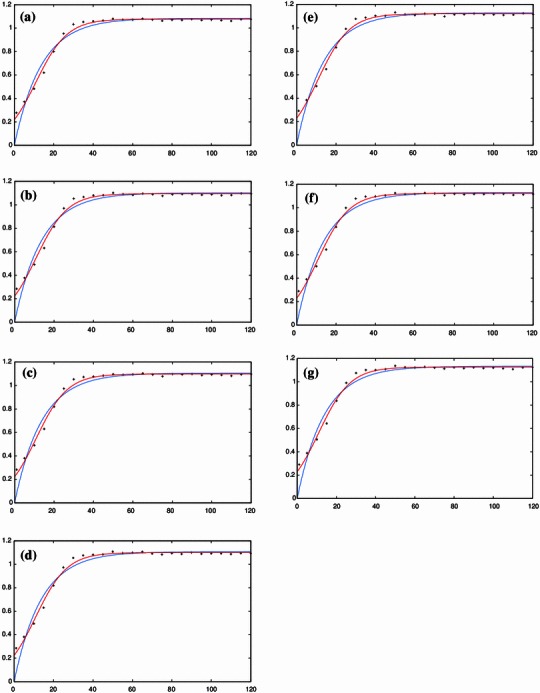
Temporal evolution of the effect of pre-leaching time (independent variable) on the yield of mangiferin content extracted from *Curcuma amada* at various experimental design points (*x* axis: time in s; *y* axis: yield in mg/g). The experimental data (*symbols*) fitted to the 2-parameter model (Eq. 1, *blue*) and 3-parameter logistic model (Eq. 2, *red*) are shown in Fig. 5a–g for 1, 5, 10, 15, 20, 25 and 30 min respectively

Figure 5a–g shows the effect of pre-leaching time on the yield of mangiferin content. Similar to Figs. 2a–e and 4a–f, the symbols represent the experimental data and the continuous curves represent the model fit. Pre-leaching time can be defined as the contact time between sample matrix and extracting solvent before microwave extraction. MAE of 2.5 g of dry *Curcuma amada* powder is carried out at microwave power of 550 W, 80 % ethanol concentration and irradiation time of 1–120 s for different pre-leaching time of 1–30 min. It is observed from these figures that with an increase in pre-leaching time from 1 to 20 min, there is an increase in mangiferin content. Beyond a pre-leaching time of 20 min, there is no noticeable increase in the yield of mangiferin content. It can be inferred that pre-leaching time of 20 min allows sufficient swelling of the plant matrix. This increased hydrated status of plant material helps in the bursting of the cell wall due to internal thermal stress and enlargement of the cellular pores, thus facilitating leaching of the target analyte. The results for ANOVA for pre-leaching time and extraction time as independent factors are given in Table 3. The Newman–Keuls test indicates that the pre-leaching time is not a significant factor contributing to the mangiferin content.

**Table 3:** Test of between-subjects effects: a two-way analysis of variance with pre-leaching time and extraction time as independent factors

Source		Type III sum of squares	*df*	Mean square	*F*	Sig.	Noncent. parameter	Observed power^a^
Pre-leaching time	Hypothesis error	3.711E−02	6	6.186E−03	254.053	0.0	1,524.317	1.0
		3.506E−03	144	2.435E−05^b^				
Extraction time	Hypothesis error	10.311	24	0.430	17,644.599	0.0	42,347.037	1.0
		3.506E−03	144	2.435E−05^b^				
Pre-leaching time × extraction time	Hypothesis error	166.914	1	166.914	388.523	0.0	388.523	1.0
		10.311	24	0.430^c^				

Dependent variable: concentration of extracted mangiferin

^a^Computed using alpha = 0.05

^b^MS (Error)

^c^MS (TMIN)

**Fig. 6:**
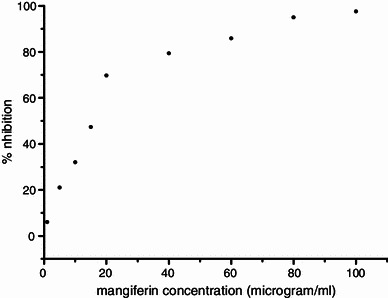
DPPH radical scavenging activity of mangiferin obtained from microwave-assisted extraction of *Curcuma amada* at microwave power—550 W, pre-leaching time—20 min, extraction time—50 s and ethanol concentration—80 %

Figure 6 shows the DPPH radical scavenging activity of mangiferin extracted from *Curcuma amada* by MAE at the optimal condition of microwave power 550 W, pre-leaching time 20 min, extraction time 50 s and ethanol concentration 80 %. It is observed that the IC50 value for mangiferin extracted from *Curcuma amada* was 17.04 μg/ml and the radical scavenging activity was directly proportional to the concentration of mangiferin with an inhibition of 97.65 % at 100 μg/ml. From this observation, it is clear that mangiferin obtained from MAE at 550 W, pre-leaching time of 20 min, extraction time of 50 s and 80 % ethanol concentration retained its antioxidant property. It is important to note here that Stoilova et al. (2005) had earlier established the antioxidant properties of mangiferin standard using DPPH radical scavenging activity of mangiferin.

**Fig. 7:**
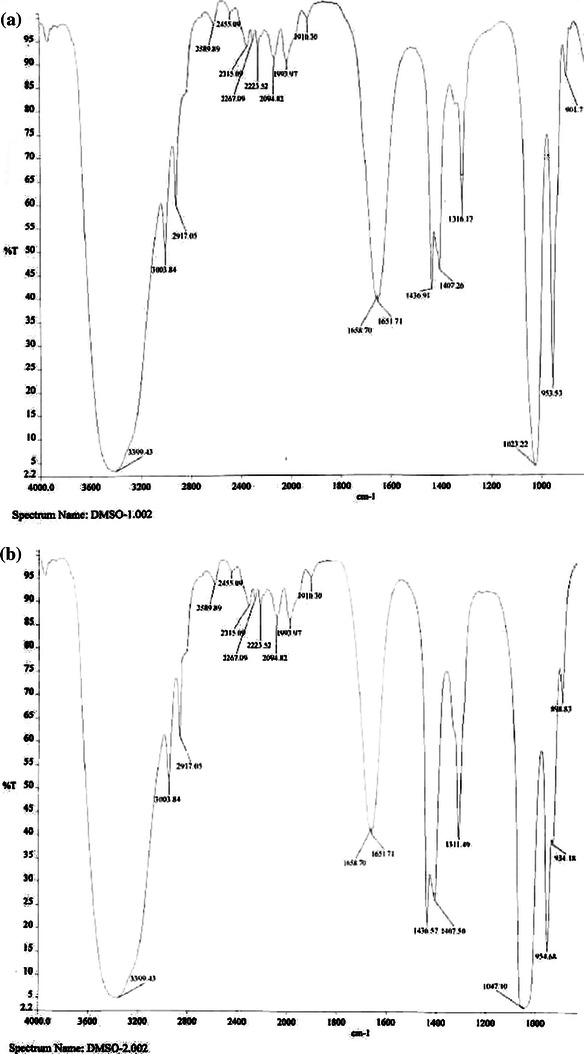
FTIR spectrum of mangiferin extracted from **a** Curcuma amada by MAE at 550 W and **b** mangiferin standard (for peak values refer Table 1)

FTIR analysis has proven to be a valuable tool for the characterization and identification of compounds or functional groups (chemical bonds) present in an unknown mixture of plant extracts (Eberhardt et al. 2007; Hazra et al. 2007). Figure 7 shows the FTIR spectrum of mangiferin extracted from *Curcuma amada* by MAE at 550 W and mangiferin standard. Six functional groups were identified: FTIR spectrum results of mangiferin after MAE showed peaks at 3,399 cm^−1^ and indicated the presence of secondary OH^−^ bond, peak at 2,917 cm^−1^ showed the presence of C–H anti-symmetric stretching, peak at 1,658.70 cm^−1^ indicated the presence C–O stretching, peak at 1,436.91 cm^−1^ indicated the presence of CH–CH bending and peak at 1,316.17 cm^−1^ indicated the presence of C–O bond. Peak at 1,023.22 cm^−1^ showed the presence of C–C stretching in the mangiferin structure. Comparing the FTIR analysis of mangiferin extracted by MAE and mangiferin standard (see Table 4) revealed the similarity and variation in the functional group. The absorption spectra showed that the C–O bond and C–O–C stretching of the mangiferin were affected during the extraction process.

**Table 4:** FTIR peak values of mangiferin after microwave-assisted extraction at 550 W with standard mangiferin

Functional group	Wave number (cm^−1^) mangiferin extracted from *Curcuma amada* by microwave-assisted extraction at 550 W	Wave number (cm^−1^) mangiferin standard
O–H	3,399.43	3,399.43
C–H	2,917.05	2,917.05
(>C=O)	1,658.70	1,660.91
C=C	1,436.91	1,436.57
C–O	1,316.17	1,311.49
C–O–C	1,023.22	1,047

**Fig. 8:**
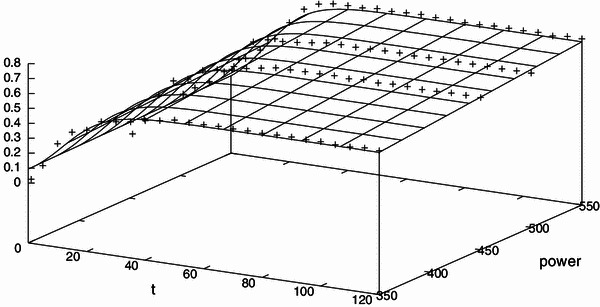
Temporal evolution of yield on microwave power during the extraction process (in accordance with a modified logistic expression; see Eq. 3) for all the design points used in the experimental setup

**Fig. 9:**
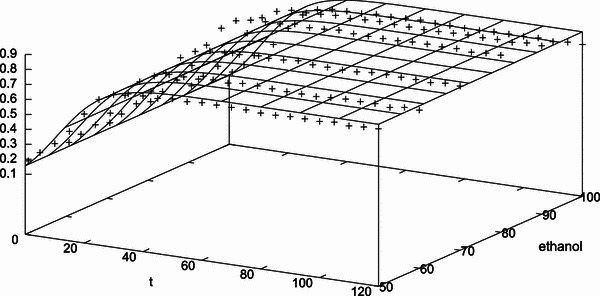
Temporal evolution of yield on the ethanol concentration during the extraction process (in accordance with a modified logistic expression; see Eq. 3) for all the design points used in the experimental setup

The results of the validation using the two-parameter first-order kinetic model (Eq. 1) and using the three-parameter logistic model (Eq. 2) for microwave power (Fig. 2a–e), ethanol concentration (Fig. 4a–f) and pre-leaching time (Fig. 5a–g) are shown, respectively. As the response for 900 W was found to vary widely from the initial five responses (i.e. 250, 350, 450, 500 and 550 W) and did not follow a clear sequence, it was neglected while validating the kinetic model for microwave power with extraction time. To check the goodness of fit, the ratio of the root mean square (RMS) value to the maximum (limit) value of mangiferin content is considered. The optimized parameter set and the corresponding value of the statistical indicator *Y*_RMS_/*Y*_max_ are summarized in Tables 5 and 6. The goodness of fit statistical indicator helps to determine how well the curve fits the data. The curve fits (based on Eq. 3) of the temporal evolution of yield on microwave power and ethanol concentration are shown in Figs. 8 and 9, respectively. The best-fit parameter values for (*P*_ref_, *k*_m_, *τ*) are found to be (759.42, 0.14, 11.68) and (2,315.22, 0.13, 10.91) using the NLLS Marquardt–Levenberg algorithm. The corresponding indicator *Y*_RMS_/*Y*_max_ equals 0.054598 and 0.070991, respectively, for microwave power and ethanol concentration, indicating a good fit.

**Table 5:** Optimized parameter set using the two-parameter modified first order kinetic model (Eq. 1) and the corresponding statistical ratio of the experimental design points for the three independent variables: microwave power, ethanol concentration and pre-leaching time

Independent variables	Experimental design points	Model parameters		Statistical indicator
		*Y*_max_ (mg/g)	*k*_m_ (s^−1^)	*Y*_RMS_/*Y*_max_ (−)
Microwave power	250 W	0.013715	0.094507	0.084504
	350 W	0.568469	0.069558	0.018957
	450 W	0.622861	0.069927	0.018408
	500 W	0.643451	0.065034	0.051907
	550 W	0.718277	0.073040	0.052717
Ethanol concentration	50 %	0.754853	0.073097	0.052993
	60 %	0.761361	0.072713	0.052911
	70 %	0.804594	0.072975	0.052548
	80 %	0.890682	0.073307	0.052608
	90 %	0.831694	0.073287	0.053215
	100 %	0.718277	0.073040	0.052717
Pre-leaching time	1 min	1.083663	0.072867	0.052288
	5 min	1.102309	0.073028	0.052639
	10 min	1.105546	0.072777	0.052213
	15 min	1.107016	0.072974	0.052566
	20 min	1.127495	0.072855	0.052249
	25 min	1.129510	0.072919	0.052386
	30 min	1.133223	0.072485	0.052057

**Table 6:** Optimized parameter set using the three-parameter delayed logistic model (Eq. 2) and the corresponding statistical ratio of the experimental design points for the three independent variables: microwave power, ethanol concentration and pre-leaching time

Independent variables	Experimental design points	Model parameters			Statistical indicator
		*Y*_max_ (mg/g)	*k*_m_ (s^−1^)	*τ* (s)	*Y*_RMS_/*Y*_max_ (−)
Microwave power	250 W	0.013716	0.013716	9.078383	0.064274
	350 W	0.558402	0.156855	11.625592	0.040377
	450 W	0.611637	0.158482	11.513974	0.040776
	500 W	0.636598	0.121393	12.340408	0.023855
	550 W	0.713695	0.125821	10.917342	0.021411
Ethanol concentration	50 %	0.749934	0.126824	10.938109	0.022006
	60 %	0.756452	0.125319	10.96249	0.021600
	70 %	0.799469	0.125387	10.909826	0.021116
	80 %	0.885108	0.126128	10.886925	0.021164
	90 %	0.826444	0.126240	10.88443	0.021859
	100 %	0.713695	0.125821	10.917342	0.021411
Pre-leaching time	1 min	1.076805	0.125075	10.928752	0.020460
	5 min	1.095355	0.125485	10.911725	0.020907
	10 min	1.098343	0.125436	10.941557	0.020672
	15 min	1.100119	0.125104	10.919032	0.020930
	20 min	1.120260	0.124949	10.905220	0.020455
	25 min	1.122212	0.125592	10.921825	0.021031
	30 min	1.126029	0.124124	10.972533	0.020265

## Conclusions

Mangiferin was extracted from *Curcuma amada* using microwave-assisted extraction technique. Maximum mangiferin content of 1.1156 mg/g was obtained at microwave power of 550 W and extraction time of 50 s with 80 % ethanol as a solvent and pre-leaching time of 20 min and retained its antioxidant properties. The experimental profiles fitted into a two-parameter modified first-order kinetic model and a three-parameter modified logistic model with sufficient accuracy. The MAE of mangiferin from *Curcuma amada* using ethanol can be safely employed in food and medicinal industries as it is not only efficient from the industrial point of view, but also eco-friendly since it prevents environmental hazards. This indicates the usefulness and significance of MAE as a novel extraction technique in biotechnological applications.

## Abbreviations

ANOVA: Analysis of variations
MAE: Microwave-assisted extraction
OFAT: One-factor-at-a-time
HPLC: High performance liquid chromatography
FTIR: Fourier transform infrared spectroscopy
SCFE: Supercritical fluid extraction
UAE: Ultrasound assisted extraction
HRE: Heat reflux extraction
